# Wide-Scope Target and Suspect Screening of Antibiotics in Effluent Wastewater from Wastewater Treatment Plants in Europe

**DOI:** 10.3390/antibiotics12010100

**Published:** 2023-01-06

**Authors:** Kelsey Ng, Nikiforos A. Alygizakis, Nikolaos S. Thomaidis, Jaroslav Slobodnik

**Affiliations:** 1 Environmental Institute, Okružná 784/42, 97241 Koš, Slovakia; 2 RECETOX, Faculty of Science, Masaryk University, Kotlářská 2, 60200 Brno, Czech Republic; 3 Laboratory of Analytical Chemistry, Department of Chemistry, National and Kapodistrian University of Athens, Panepistimiopolis Zografou, 15771 Athens, Greece

**Keywords:** antibiotics, wide-scope target screening, wide-scope suspect screening, ecotoxicological risk assessment, antibiotic-resistance

## Abstract

The occurrence of antibiotics in the environment could result in the development of antibiotic-resistant bacteria, which could result in a public health crisis. The occurrence of 676 antibiotics and the main transformation products (TPs) was investigated in the 48 wastewater treatment plants (WWTPs) from 11 countries (Germany, Romania, Serbia, Croatia, Slovenia, Hungary, Slovakia, Czechia, Austria, Cyprus, and Greece) by target and suspect screening. Target screening involved the investigation of antibiotics with reference standards (40 antibiotics). Suspect screening covered 676 antibiotics retrieved from the NORMAN Substance Database (antibiotic list on NORMAN network). Forty-seven antibiotics were detected in effluent wastewater samples: thirty-two by target screening and fifteen additional ones by suspect screening. An ecotoxicological risk assessment was performed based on occurrence data and predicted no effect concentration (PNEC), which involved the derivation of frequency of appearance (FoA), frequency of PNEC exceedance (FoE), and extent of PNEC exceedance (EoE). Azithromycin, erythromycin, clarithromycin, ofloxacin, and ciprofloxacin were prioritized as the calculated risk score was above 1. The median of antibiotics’ load to freshwater ecosystems was 0.59 g/day/WWTP. The detection of antibiotics across countries indicates the presence of antibiotics in the ecosystems of Europe, which may trigger unwanted responses from the ecosystem, including antibiotic resistance.

## 1. Introduction

Antibiotics have been extensively used by human in various ways; on top of medical use, they are also employed in agriculture, aquaculture, animal husbandry, horticulture, food preservation, and industries such as ethanol production [1]. This entails abuse of antibiotics in certain sectors such as medical use in humans without prescription. The heavy use of antibiotics, which includes certain misuse, would lead to heavy discharge of antibiotics in wastewater and consequently a consistent increment in the amount of antibiotics in the environment [2]. The increasing abundance of antibiotics in ecosystems would affect the natural microbial communities by promoting antibiotic resistance in bacteria [3].

Under exposure to antibiotics, bacteria which manage to survive would be able to proliferate and to pass the resistant traits to succeeding generations [4]. Antibiotic resistance, as an illustration of natural selection, can be acquired through horizontal gene transfer or mutation [5]. It was demonstrated that antibiotic consumption is directly related to the rise in resistant strains in bacteria [6]. As warned by Sir Alexander Fleming, Nobel Prize winner in 1945 for the discovery of penicillin, the abuse of antibiotics would promote resistant bacteria [7]. The development of antibiotic resistance as a result of antibiotic overuse has been observed across the globe [8,9].

Without proper wastewater treatment, the disposal of antibiotics could lead to the development of bacteria that is not susceptible to antibiotics, superbugs, which could result in serious public health crisis followed by thousands of deaths [10,11]. In light of the situation, WHO listed antibiotic resistance as one of the biggest threats to global health, food security, and development [12]. The situation can be alleviated by the elimination of the abuse of antibiotics and proper removal of antibiotics from wastewater [13]. Being one of the most common wastewater treatment systems, the conventional activated sludge (CAS) system is adopted in majority of the studied WWTPs. CAS systems are generally well designed for removing organic compounds and nutrients, but the removal variability on certain antibiotics is large, which could promote the development of antibiotic-resistance in the aquatic ecosystems [2,14]. Therefore, it is essential to establish the occurrence profile of antibiotics in wastewater, which could be used for the forecast of antibiotic resistance utilizing artificial intelligence and statistical approaches [15].

**Figure 1:**
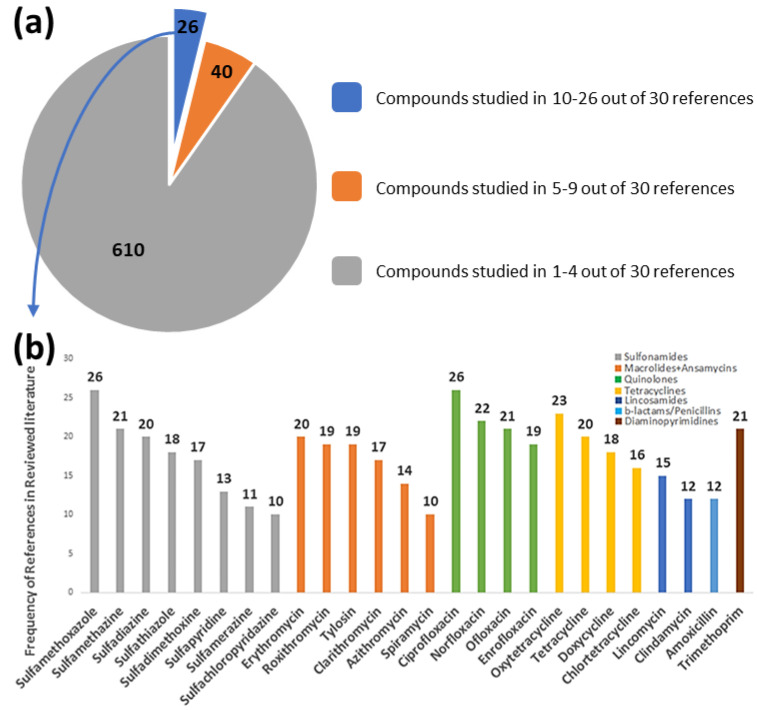
(**a**) Pie chart representing the availability of occurrence profile of the 676 studied antibiotics from the 30 references in the literature review; (**b**) bar chart showing frequency of references (and chemical classes by colour) for the 26 most studied antibiotics (blue shaded in the pie chart).

An extensive literature review was conducted to select a set of antibiotics to be studied in effluent wastewater samples, which is available to the scientific community on the NORMAN Suspect List Exchange as the List S6 ITNANTIBIOTIC [16]. A smaller scale literature review (30 previous studies on antibiotic occurrence) revealed that few occurrence data were established for over 600 among the 676 selected antibiotics, as shown in the grey shaded area of Figure 1a. Only 26 of the investigated antibiotics are frequently studied in the 30 references of the literature review, as indicated in Figure 1b. In order to fill in this gap in the occurrence profile of antibiotics, wide-scope suspect screening of antibiotics and TPs was performed. Environmental samples were analyzed by liquid chromatography coupled with high-resolution mass spectrometry (LC-HRMS), which is a powerful tool to investigate the occurrence of antibiotics and their TPs via wide-scope screening of suspected antibiotics without reference standards.

The aims of the study were (1) to screen for antibiotics in the effluent wastewater samples with a special focus on the less studied ones; (2) to establish the occurrence profile of antibiotics across European countries; and (3) to perform ecotoxicological risk assessment and prioritization of the detected antibiotics. The ultimate goal of this study was to investigate the emission profile of antibiotics from WWTPs in Europe.

## 2. Results and Discussion

### 2.1. Wide-Scope Target and Suspect Screening and Risk Assessment

Target screening identified 32 antibiotics in the wastewater. Five of them were prioritized as they showed risk score above 1: three macrolides (azithromycin, erythromycin. and clarithromycin) and two quinolones (ofloxacin and ciprofloxacin). Some of the prioritized antibiotics of environmental concern have been characterized in previous studies. Azithromycin and ciprofloxacin were proposed as markers of antibiotic pollution for the analysis of effluent wastewater [17]. Clarithromycin and ofloxacin were prioritized in the characterization of effluent wastewater from 12 WWTPs of Danube River Basin (DRB) countries in 2019 [18].

Wide-scope suspect screening of antibiotics was performed to avoid overlooking analytes, which successfully detected 15 antibiotics overlooked by target screening. Most of these compounds were not screened for by the conventional target screening approach in previous studies of antibiotics in WWTP effluents [17,19,20]. Table 1 demonstrated the risk assessment results on the 47 detected antibiotics (by target and suspect screening). It is urgent to tackle the issue by properly removing the prioritized antibiotics from WWTP to avoid further discharge of these chemicals into the environment.

Azithromycin is one of the most commonly used antibiotics and is frequently found in WWTP owing to its low metabolic rate [21] and long half-life [22]. The presence of azithromycin in wastewater could result in the development of bacterial resistance owing to its broad-spectrum of antimicrobial activity [21]. UV radiation with sodium persulfate was found to be a reliable tool to remove azithromycin from wastewater—as high as 99% degradation [23].

The common sewage treatment process was not able to remove erythromycin and its main degradation products [24] from reclaimed water. These compounds accumulated in soil for over 5 months, which allows time to develop resistance in bacteria [25]. WWTP mediated by the microalgae-bacteria consortium was able to remove over 90% erythromycin from wastewater [26].

Clarithromycin in wastewater posed a risk as it would aid the development of resistance in multiple bacteria including *Helicobacter pylori* [27]. A novel electro-Fenton process [28] was able to remove over 99% clarithromycin from wastewater originated from medical laboratories.

Ofloxacin and ciprofloxacin both pose significant environmental risk due to their genotoxicity [29,30], and they also exhibit resistant to microbial degradation [31,32]. A metal-organic framework material was found to be a powerful tool that is able to remove over 95% of ofloxacin from wastewater samples [33]. Ozonation was able to remove over half of ofloxacin and ciprofloxacin from untreated wastewater and to completely remove them from treated wastewater with lower concentrations [34].

Despite having a risk score below 1, some antibiotics were detected in over half of the wastewater samples studied, including sulfapyridine, trimethoprim, lincomycin, flumequine, metronidazole, sulfamethoxazole, oxolinic acid, and amoxicillin by target screening and 8-hydroxyquinoline, clindamycin, linezolid, fluconazole, sulfasalazine, mycophenolic acid, roxithromycin, and cordycepin by suspect screening. Target screening of these suspected antibiotics would enhance the robustness of the concentration determination, which is essential for checking for PNEC exceedance. The majority of these antibiotics were detected below P-PNEC. The frequency of PNEC exceedance (FoE) of these antibiotics was between 0 and 0.01, which resulted in a risk score below 1. The derivation of robust EQS PNEC values is important to verify that these antibiotics at detected levels do not pose threats to the ecosystem.

Antibiotics are commonly used together in synergistic antimicrobial combination (also known as combination therapy) for treating bacterial or fungal infections. A common pair is trimethoprim and sulfamethoxazole [35], which were detected in over 50% of the sampled sites in target screening. Synergistic antimicrobial combination could provide an antimicrobial effect even when resistance has developed for a single agent [36,37]. This fact makes it popular in medical use, resulting in high loads of antibiotics in WWTPs. Nevertheless, studies revealed that combination therapy imposes selective pressures in the microbial community, promoting the evolution of multi-drug resistance [38,39]. The benefits of combination therapy could be lost in a few bacterial generations, resulting in the development of highly infectious pathogens [40]. The high FoA of multiple antibiotics in the sampled sites could result in such an evolution of multi-drug resistance in the microbial community. An investigation into the occurrence of antibiotic-resistant genes in the studied sites could reveal the impacts of antibiotic pollution. Future effect-based studies utilizing bioanalytical tools such as in vitro bioassays could reveal the other adverse effects on the ecosystems caused by the wide-spread occurrence of antibiotics [18].

### 2.2. Loads of Antibiotics into Freshwater Ecosystems

**Figure 2:**
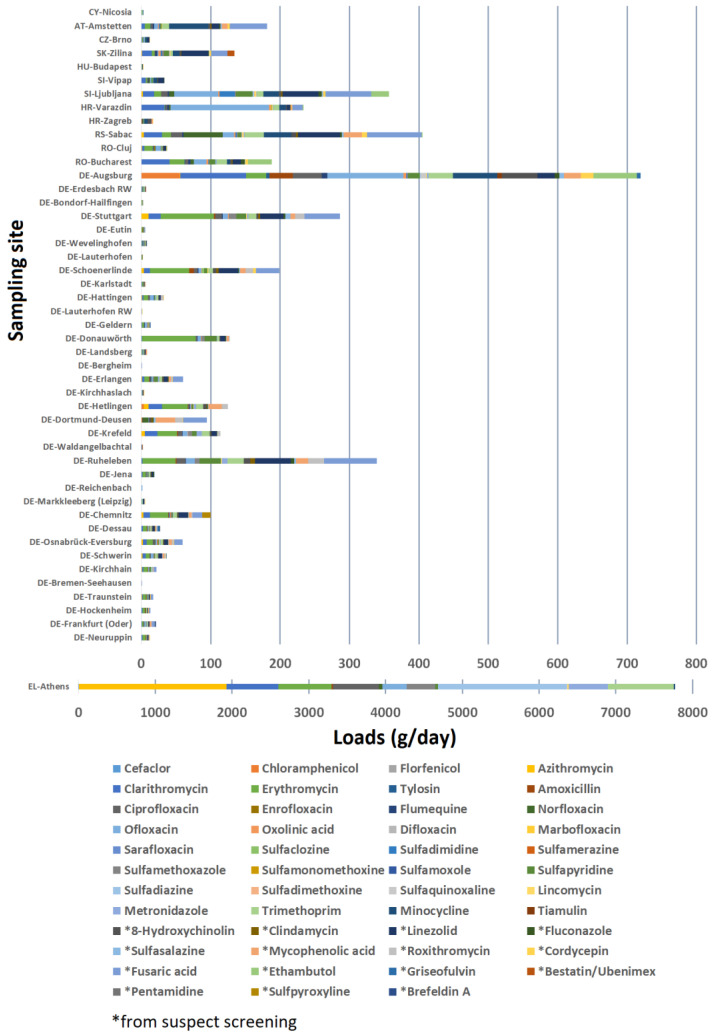
Stacked bar plot of antibiotic loads into freshwater ecosystems expressed in gram per day (Germany DE, Romania RO, Serbia RS, Croatia HR, Slovenia SI, Hungary HU, Slovakia SK, Czechia CZ, Austria AT, Cyprus CY, Greece EL).

Figure 2 illustrated the amount of studied antibiotics entering freshwater ecosystems through effluent wastewater discharge at the sampling sites. The loads were obtained as the product of the daily flow rate of the WWTP and the concentration of the detected antibiotics from chemical screening.

Total antibiotic load per WWTP spanned from below 1 g/day to over 7000 g/day, which was a result of the broad range of scale of the WWTPs—small WWTPs such as the ones in Bremen–Seehausen and Bergheim serve less than 10,000 people, which resulted in less than 1 g/day of total antibiotics load, while large WWTPs such as the Athens WWTP serves over 3.7 million people, which resulted in 7000 g/day of total antibiotics load. The majority of the small WWTPs would load the effluent wastewater (together with antibiotics) to river water ecosystems, while the large WWTPs such as the one in Athens would load the effluent water with antibiotics to sea water ecosystems, which gives a much higher dilution factor to the final concentration of antibiotics that exist in the destination. Therefore, it would be misleading to compare just the total antibiotic load between WWTPs without considering the flow rates and the disposal ecosystem.

Germany showed a general total antibiotic load profile of below 100 g/day in the majority of WWTPs, which was an order of magnitude lower than WWTPs of similar flow rates across DRB countries such as the ones in Varazdin, Ljubljana, and Zilina. This fact suggested a possible better performance in antibiotic removal for the German WWTPs. Sabac was among the top three WWTPs on daily loads, which is consistent with previous finding of high antibiotic release at the area due to the local pharmaceutical industry (production of erythromycin, sulfamethoxazole, and ciprofloxacin) [18]. Nevertheless, only one to two WWTPs were investigated for each DRB country, which were insufficient to conclude the loads of antibiotics from WWTPs in these countries. A wide scope study on more WWTPs in these countries would give a more comprehensive profile of the antibiotic loads and yield a comparison in antibiotic loads between countries.

German WWTPs showed a similar pattern in which erythromycin accounted for 14–60% of the loads in 28 out of 35 of the German WWTPs studied. This further proves the need for proper removal for erythromycin (a prioritized antibiotic) in wastewater, especially in Germany. Erythromycin, azithromycin, clarithromycin, sulfadiazine, and trimethoprim revealed loads of > 1 kg/day across the 48 WWTPs. These antibiotics can potentially be used as additional indicators when optimizing the wastewater performance. The median total load (sum of loads in 48 WWTPs for a given antibiotic) was 35.9 g/day, while the median individual load per WWTP for a given antibiotic was 0.59 g/day. Advanced post treatment following the CAS system (adopted in majority of the studied WWTPs) could significantly enhance the removal of antibiotics, namely UV and ozonation [41].

Despite the high amounts of antibiotics entering freshwater ecosystems through effluent wastewater discharge which could accelerate the evolution of bacteria into superbugs, there is not yet a regulatory system to control the environmental risk caused by these compounds. Certain methodologies had been proposed for setting the regulatory limits based on PNECs, including the derivation of microbial system-specific minimal selective concentrations (MSCs) [42,43], and emission limit values (ELVs), which adopted a safety factor of five to give the equation ELV = 5 × PNEC [44]. There is an urgent need for the establishment of a regulatory emission limit, which was reflected in the high loads of antibiotics found in this study. Nonetheless, a larger scale wide-scope screening of antibiotics (investigation on more WWTPs in each country) is required to establish the national antibiotic load profile.

## 3. Materials and Methods

### 3.1. Investigated Samples

**Figure 3:**
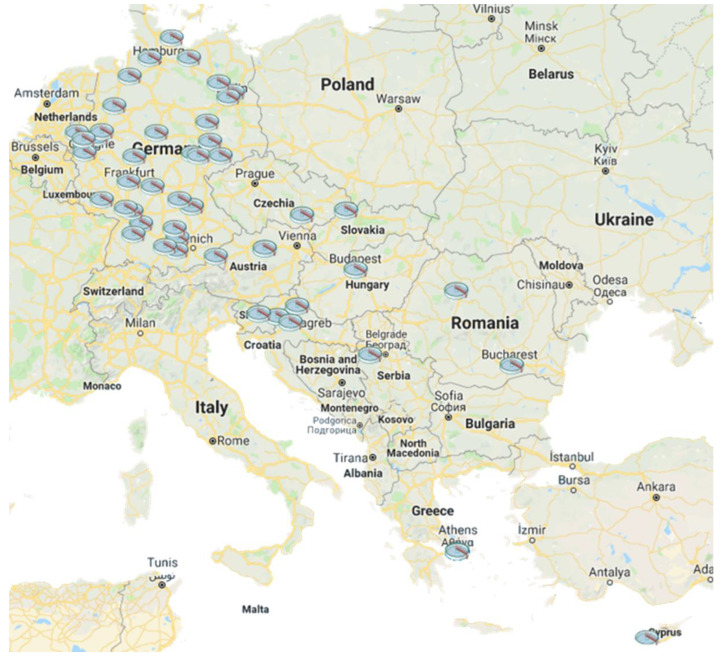
Spatial distribution of the 48 sampled WWTPs. The WWTPs were located in 11 European countries: Germany, Romania, Serbia, Croatia, Slovenia, Hungary, Slovakia, Czechia, Austria, Greece and Cyprus.

Effluent wastewater samples were collected from various projects from August 2017 to October 2018 [18,45,46], and their spatial distribution is shown in Figure 3. In cooperation with the German Federal Agency, 34 WWTP samples were collected in Germany in May 2018 [45], whereas the second set of samples included twelve WWTPs in nine DRB countries and was collected during the effluent wastewater sampling campaign of August 2017 in cooperation with the International Commission for the Protection of Danube River [18]. WWTP samples from Athens were collected during the sampling campaign of March 2018, which is one of the biggest WWTPs in Europe [46]. Sampling sites also included the WWTP of Nicosia in Cyprus. Flow-proportional samples were collected in all cases.

### 3.2. Sample Preparation and Instrumental Analysis

After collection, samples were stored in the WWTP at −20 °C and remained frozen during transportation. Effluent water samples were cleaned up and pre-concentrated 4000 times on an Atlantic HLB-M Disk using HORIZON SPE-DEX 4790 (USA) with a 47 mm disk holder, in accordance with the extraction programme presented elsewhere [18].

### 3.3. Quality Assurance and Quality Control

The chemical method used in the study was validated in terms of linearity, sensitivity, accuracy, and repeatability [18,45]. Seven-point calibration curves were generated using linear regression analysis in the range of 0.5–1000 ng L^−1^, and a linear correlation coefficient (R^2^) was used to assess the linearity. R^2^ was proved to be equal to or higher than 0.99 for all targeted substances.

Regarding accuracy, the recovery of 10.0 and 100.0 ng L^−1^ standards in effluent wastewater samples was studied. The extraction recovery was evaluated by looking at the ratio between peak area of spiked samples and that of matrix-matched samples. Extraction recovery was calculated by dividing the peak area of the spiked samples by the peak area of the matrix-matched samples spiked at the end of the sample preparation. As target compounds may be present in actual samples, the difference between readings from wastewater, spiked, and matrix-matched samples was used to determine the analyte concentration. In total, 22 compounds showed recovery of 80–120%, 11 compounds showed recovery of 60–80%, and 7 compounds showed poor recovery of below 60%. The method repeatability was measured by intermediate precision, where the relative standard deviation (RSD, established by dividing the standard deviation value by the mean value of the measured concentration) at 10.0 and 100.0 ng L^−1^ was measured and was proved to be below 20% for all targets. The limit of detection (LOD) was found to be in the range 0.02–6.1 ng L^−1^. For quality assurance purposes, the samples were spiked with a list of internal standards to ensure satisfactory extraction and instrumental analysis. To check for contamination, laboratory procedural blanks and field blanks were used, which underwent chemical analysis together with the wastewater samples. The difference between signals from samples and blanks was adopted for the screening.

Level of confidence of the identification was expressed in levels from 1 to 5, where 5 denotes the exact mass(es) of interest (lowest confidence). Level 4 entails unequivocal molecular formulas; level 3 indicates tentative candidates; level 2 suggests probable structures with diagnostic evidence; and level 1 implies confirmed structures with reference standards [47]. Detailed information on the quality assurance and quality control aspects can be found elsewhere [18,45].

### 3.4. Suspect Screening and Semi-Quantification Analysis

To avoid overlooking antibiotics, all samples were screened for the suspect list of antibiotics and their major TPs (List S6 ITNANTIBIOTIC on NORMAN Suspect List Exchange [16,48]) using the NORMAN Digital Sample Freezing Platform (DSFP) [49]. In suspect screening, the retention time index (RTI) and MS fragmentation prediction of the target compounds were established with the Development and Prediction of Retention Time Indices for LC-HRMS (version 2.5.0) [50] and CMF-ID software [51], respectively, based on the chemical structure of the antibiotic or TP of interest. LC-HRMS chromatograms of the environmental samples were compared to the RTI and MS fragmentation prediction of the antibiotics and TPs studied in an automated manner.

Suspected antibiotics were semi-quantified based on the target compound with the highest structural similarity, using the standard addition calibration curve of the target compound in spiked effluent wastewater [52]. For instance, roxithromycin (a suspected macrolide antibiotic) was semi-quantified based on the calibration curve of azithromycin (another macrolide antibiotic), where the two compounds share structural similarity of 71%. Based on the atom pairs and sequences, 2D-linear fragment descriptors were calculated [53] and the Tanimoto coefficient was used to determine the structural similarity between compounds. The semi-quantification methodology was validated with 778 compounds on a wide spectrum of physicochemical properties in both negative ionization (207 compounds) and positive ionization (681 compounds) [52]. Calibration curves previously used for quantification in target screening were used to semi-quantify structurally similar suspected compounds. The degree of structural similarity could be used to evaluate the accuracy of the semi-quantification, where a higher similarity score entailed lower uncertainty. A risk assessment of the wide-scope suspect screening was based on the semi-quantified concentrations of the suspected compounds, using the same methodology applied to the risk assessment on target screening.

### 3.5. Risk Assessment and Prioritization

The risk assessment was performed on the detected target and suspect antibiotics based on the prioritization methodology established by the NORMAN [54,55]. The method entails a comparison between detected concentration of analytes with the PNEC, which denotes the ecotoxicological threshold value. In cases in which no experimental data on the PNEC of the analyte were available, predicted PNECs (P-PNECs) were derived by the QSTR models [56]. For risk assessment purposes, the lowest PNEC was selected in the order of (a) environmental quality standards (EQS); (b) experimental PNEC values from reference laboratories; and (c) in silico predicted PNEC.

Risk score was established as the sum of three indicators (0–1 for each indicator): (i) Frequency of Appearance (FoA); (ii) Frequency of PNEC Exceedance (FoE); and (iii) Extent of PNEC Exceedance (EoE). FoA considers the frequency of monitoring sites where the analyte was detectable (above LOD). FoE denotes the frequency of sampled sites with maximum observations–maximum environmental concentration (MEC_site_) of an analyte above the lowest PNEC. EoE represents the ratio between the 95th percentile of all MEC_site_ values per analyte (MEC_95_) and the PNEC of the analyte, which shows the extent of the effects expected. The risk score was established as the sum of FoA, FoE, and EoE, each scaled from 0 to 1. For prioritization purposes, analytes with a risk score over 1 were prioritized. The remaining substances were considered to pose less risk to the ecosystems. Further details of the prioritization scheme could be found elsewhere [57].

## 4. Conclusions

Wide-scope screening of 676 antibiotics and its main TPs in the effluent wastewater samples in 11 countries across Europe resulted in the detection of 47 antibiotics, of which 32 were detected by target screening and 15 additional ones were detect by suspect screening. Azithromycin, erythromycin, clarithromycin, ofloxacin, and ciprofloxacin were prioritized as they each entailed risk score of above 1. These ranked compounds are of regulatory interest.

Sulfapyridine, trimethoprim, lincomycin, flumequine, metronidazole, sulfamethoxazole, oxolinic acid, and amoxicillin detected in target screening and 8-hydroxyquinoline, clindamycin, linezolid, fluconazole, sulfasalazine, mycophenolic acid, roxithromycin, and cordycepin detected in suspect screening all showed FoA over 0.5, indicating that each of them were detected in over half of the WWTPs investigated. They were widespread in wastewater in European countries. Target screening of these suspected antibiotics would provide a more robust quantification for risk assessment. Wide-scope screening of antibiotics and TPs in a wider range of WWTPs across Europe would reveal more information on the occurrence profile of these chemicals across the continent and “hotspots” of antibiotic contamination, which is essential to develop a regulatory monitoring framework on antibiotics and TPs in Europe. An investigation into the occurrence of antibiotic-resistant genes and effect-based assessment at aquatic ecosystems is recommended to reveal the impacts of antibiotic contamination, especially at “hotspots” that are highly polluted by antibiotics.

Total antibiotic load per WWTP spanned from below 1 g/day to over 7000 g/day, which was related to the population that the WWTP serves and its flow rate. Over 1 kg each of azithromycin, clarithromycin, erythromycin, sulfadiazine, and trimethoprim are discharged into freshwater ecosystems every day from the 48 WWTPs investigated in total. The median total load for a given antibiotic across the 48 WWTPs was 35.85 g/day, while the median individual antibiotic load per WWTP was 0.59 g/day. The high antibiotic load reflected the urgent needs for the establishment of regulatory limits on the emission of antibiotics.

## Figures and Tables

**Table 1:** Antibiotics detected in effluent wastewater samples (from target and suspect screening).

Antibiotic	InChIKey	Antibiotics Class	FoA	FoE	EoE	Risk Score
**Ofloxacin**	GSDSWSVVBLHKDQ-UHFFFAOYSA-N	Quinolones	1.00	0.85	1.00	**2.85**
**Azithromycin**	MQTOSJVFKKJCRP-BICOPXKESA-N	Macrolides	0.83	0.63	0.43	**1.89**
**Erythromycin**	ULGZDMOVFRHVEP-RWJQBGPGSA-N	Macrolides	0.96	0.54	0.16	**1.66**
**Clarithromycin**	AGOYDEPGAOXOCK-KCBOHYOISA-N	Macrolides	0.98	0.35	0.10	**1.43**
**Ciprofloxacin**	MYSWGUAQZAJSOK-UHFFFAOYSA-N	Quinolones	0.96	0.10	0.03	**1.09**
Sulfapyridine	GECHUMIMRBOMGK-UHFFFAOYSA-N	Sulfonamides	0.98	0.00	0.00	0.98
Trimethoprim	IEDVJHCEMCRBQM-UHFFFAOYSA-N	Dihydrofolate reductase inhibitor	0.98	0.00	0.00	0.98
* 8-Hydroxyquinoline	MCJGNVYPOGVAJF-UHFFFAOYSA-N	Other	0.91	0.00	0.00	0.91
Lincomycin	OJMMVQQUTAEWLP-KIDUDLJLSA-N	Lincosamides	0.88	0.00	0.00	0.88
Flumequine	DPSPPJIUMHPXMA-UHFFFAOYSA-N	Quinolones	0.85	0.00	0.00	0.85
* Clindamycin	KDLRVYVGXIQJDK-NOWPCOIGSA-N	Other	0.83	0.04	0.01	0.84
* Linezolid	TYZROVQLWOKYKF-ZDUSSCGKSA-N	Other	0.83	0.00	0.00	0.83
* Fluconazole	RFHAOTPXVQNOHP-UHFFFAOYSA-N	Other	0.79	0.00	0.00	0.83
Metronidazole	VAOCPAMSLUNLGC-UHFFFAOYSA-N	Other	0.81	0.00	0.00	0.81
Sulfamethoxazole	JLKIGFTWXXRPMT-UHFFFAOYSA-N	Sulfonamides	0.79	0.00	0.00	0.79
* Sulfasalazine	NCEXYHBECQHGNR-UHFFFAOYSA-N	Sulfonamides	0.79	0.00	0.00	0.79
Oxolinic acid	KYGZCKSPAKDVKC-UHFFFAOYSA-N	Quinolones	0.77	0.00	0.00	0.77
* Mycophenolic acid	HPNSFSBZBAHARI-RUDMXATFSA-N	Other	0.70	0.06	0.01	0.73
* Roxithromycin	RXZBMPWDPOLZGW-XMRMVWPWSA-N	Macrolides	0.66	0.00	0.00	0.70
Amoxicillin	LSQZJLSUYDQPKJ-NJBDSQKTSA-N	Penicillins	0.67	0.00	0.00	0.67
* Cordycepin	OFEZSBMBBKLLBJ-BAJZRUMYSA-N	Other	0.57	0.00	0.00	0.57
* Fusaric acid	DGMPVYSXXIOGJY-UHFFFAOYSA-N	Other	0.43	0.00	0.00	0.43
Sulfadimidine (Sulfamethazine)	ASWVTGNCAZCNNR-UHFFFAOYSA-N	Sulfonamides	0.42	0.00	0.00	0.42
Minocycline	DYKFCLLONBREIL-KVUCHLLUSA-N	Tetracycline	0.29	0.04	0.01	0.34
Florfenicol	AYIRNRDRBQJXIF-NXEZZACHSA-N	Amphenicols	0.29	0.00	0.00	0.29
Norfloxacin	OGJPXUAPXNRGGI-UHFFFAOYSA-N	Quinolones	0.27	0.00	0.00	0.27
Sulfadiazine	SEEPANYCNGTZFQ-UHFFFAOYSA-N	Sulfonamides	0.21	0.02	0.00	0.23
Chloramphenicol	WIIZWVCIJKGZOK-RKDXNWHRSA-N	Amphenicols	0.21	0.00	0.00	0.21
Cefaclor	QYIYFLOTGYLRGG-GPCCPHFNSA-N	Cefalosporines	0.21	0.00	0.00	0.21
Sulfamerazine	QPPBRPIAZZHUNT-UHFFFAOYSA-N	Sulfonamides	0.21	0.00	0.00	0.21
Tylosin	WBPYTXDJUQJLPQ-VMXQISHHSA-N	Macrolides	0.17	0.00	0.00	0.17
Sulfaclozine	QKLPUVXBJHRFQZ-UHFFFAOYSA-N	Sulfonamides	0.17	0.00	0.00	0.17
Enrofloxacin	SPFYMRJSYKOXGV-UHFFFAOYSA-N	Quinolones	0.15	0.00	0.00	0.15
* Ethambutol	AEUTYOVWOVBAKS-UWVGGRQHSA-N	Other	0.13	0.00	0.00	0.13
Marbofloxacin	BPFYOAJNDMUVBL-UHFFFAOYSA-N	Quinolones	0.06	0.00	0.00	0.06
Sulfadimethoxine	ZZORFUFYDOWNEF-UHFFFAOYSA-N	Sulfonamides	0.06	0.00	0.00	0.06
* Griseofulvin	DDUHZTYCFQRHIY-RBHXEPJQSA-N	Other	0.06	0.00	0.00	0.06
* Bestatin/Ubenimex	VGGGPCQERPFHOB-RDBSUJKOSA-N	Other	0.06	0.00	0.00	0.06
Sarafloxacin	XBHBWNFJWIASRO-UHFFFAOYSA-N	Quinolones	0.04	0.00	0.00	0.04
Sulfamonomethoxine	WMPXPUYPYQKQCX-UHFFFAOYSA-N	Sulfonamides	0.04	0.00	0.00	0.04
Sulfamoxole	CYFLXLSBHQBMFT-UHFFFAOYSA-N	Sulfonamides	0.04	0.00	0.00	0.04
Sulfaquinoxaline	NHZLNPMOSADWGC-UHFFFAOYSA-N	Sulfonamides	0.04	0.00	0.00	0.04
Tiamulin	UURAUHCOJAIIRQ-QGLSALSOSA-N	Other	0.04	0.00	0.00	0.04
* Pentamidine	XDRYMKDFEDOLFX-UHFFFAOYSA-N	Other	0.04	0.00	0.00	0.04
Difloxacin	NOCJXYPHIIZEHN-UHFFFAOYSA-N	Quinolones	0.02	0.00	0.00	0.02
* Sulfpyroxyline	FBFBRAFXKGRRHI-UHFFFAOYSA-N	Sulfonamides	0.02	0.00	0.00	0.02
* Brefeldin A	KQNZDYYTLMIZCT-KQPMLPITSA-N	Other	0.02	0.00	0.00	0.02

* From suspect screening.

## Data Availability

The data represented in this study are available in the article.

## References

[1] Meek R.W., Vyas H., Piddock L.J. (2015). Nonmedical Uses of Antibiotics: Time to Restrict Their Use?. PLoS Biol..

[2] Jendrzejewska N., Karwowska E. (2018). The influence of antibiotics on wastewater treatment processes and the development of antibiotic-resistant bacteria. Water Sci. Technol..

[3] Grenni P., Ancona V., Caracciolo A.B. (2018). Ecological effects of antibiotics on natural ecosystems: A review. Microchem. J..

[4] Berendonk T.U., Manaia C.M., Merlin C., Fatta-Kassinos D., Cytryn E., Walsh F., Burgmann H., Sorum H., Norstrom M., Pons M.N. (2015). Tackling antibiotic resistance: The environmental framework. Nat. Rev. Microbiol..

[5] Ventola C.L. (2015). The antibiotic resistance crisis: Part 1: Causes and threats. Pharm. Ther..

[6] Datta S., Pal N.K., Nandy A.K. (2013). The antibiotic alarm. Nature.

[7] Rosenblatt-Farrell N. (2009). The landscape of antibiotic resistance. Environ. Health Perspect..

[8] Read A.F., Woods R.J. (2014). Antibiotic resistance management. Evol. Med. Public Health.

[9] Spellberg B., Gilbert D.N. (2014). The future of antibiotics and resistance: A tribute to a career of leadership by John Bartlett. Clin. Infect. Dis..

[10] Mohanty D. (2019). Rational Use of Antibiotics: Time to Join the War Against Superbugs. Indian J. Surg..

[11] Domingues C.P.F., Rebelo J.S., Pothier J., Monteiro F., Nogueira T., Dionisio F. (2021). The Perfect Condition for the Rising of Superbugs: Person-to-Person Contact and Antibiotic Use Are the Key Factors Responsible for the Positive Correlation between Antibiotic Resistance Gene Diversity and Virulence Gene Diversity in Human Metagenomes. Antibiotics.

[12] (2020). World Health Organization: Antibiotic Resistance. https://www.who.int/news-room/fact-sheets/detail/antibiotic-resistance.

[13] Paulus G.K., Hornstra L.M., Alygizakis N., Slobodnik J., Thomaidis N., Medema G. (2019). The impact of on-site hospital wastewater treatment on the downstream communal wastewater system in terms of antibiotics and antibiotic resistance genes. Int. J. Hyg. Environ. Health.

[14] Chen C.X., Aris A., Yong E.L., Noor Z.Z. (2022). A review of antibiotic removal from domestic wastewater using the activated sludge process: Removal routes, kinetics and operational parameters. Environ. Sci. Pollut. Res. Int..

[15] Elsheikh A.H., Saba A.I., Panchal H., Shanmugan S., Alsaleh N.A., Ahmadein M. (2021). Artificial Intelligence for Forecasting the Prevalence of COVID-19 Pandemic: An Overview. Healthcare.

[16] NORMAN (2021). NORMAN Suspect List Exchange—NORMAN SLE. https://www.norman-network.com/nds/SLE/.

[17] Rodriguez-Mozaz S., Vaz-Moreira I., Varela Della Giustina S., Llorca M., Barcelo D., Schubert S., Berendonk T.U., Michael-Kordatou I., Fatta-Kassinos D., Martinez J.L. (2020). Antibiotic residues in final effluents of European wastewater treatment plants and their impact on the aquatic environment. Environ. Int..

[18] Alygizakis N.A., Besselink H., Paulus G.K., Oswald P., Hornstra L.M., Oswaldova M., Medema G., Thomaidis N.S., Behnisch P.A., Slobodnik J. (2019). Characterization of wastewater effluents in the Danube River Basin with chemical screening, in vitro bioassays and antibiotic resistant genes analysis. Environ. Int..

[19] Wang K., Zhuang T., Su Z., Chi M., Wang H. (2021). Antibiotic residues in wastewaters from sewage treatment plants and pharmaceutical industries: Occurrence, removal and environmental impacts. Sci. Total Environ..

[20] Kortesmaki E., Ostman J.R., Meierjohann A., Brozinski J.M., Eklund P., Kronberg L. (2020). Occurrence of Antibiotics in Influent and Effluent from 3 Major Wastewater-Treatment Plants in Finland. Environ. Toxicol. Chem..

[21] Koch D.E., Bhandari A., Closb L., Hunter R.P. (2005). Azithromycin extraction from municipal wastewater and quantitation using liquid chromatography/mass spectrometry. J. Chromatogr. A.

[22] Walters E., McClellan K., Halden R.U. (2010). Occurrence and loss over three years of 72 pharmaceuticals and personal care products from biosolids-soil mixtures in outdoor mesocosms. Water Res..

[23] Sadeghi M., Sadeghi R., Ghasemi B., Mardani G., Ahmadi A. (2018). Removal of Azithromycin from Aqueous Solution Using UV- Light Alone and UV Plus Persulfate (UV/Na2S2O8) Processes. Iran. J. Pharm. Res..

[24] Xu W., Zhang G., Li X., Zou S., Li P., Hu Z., Li J. (2007). Occurrence and elimination of antibiotics at four sewage treatment plants in the Pearl River Delta (PRD), South China. Water Res..

[25] Kulkarni P., Olson N.D., Raspanti G.A., Rosenberg Goldstein R.E., Gibbs S.G., Sapkota A., Sapkota A.R. (2017). Antibiotic Concentrations Decrease during Wastewater Treatment but Persist at Low Levels in Reclaimed Water. Int. J. Environ. Res. Public Health.

[26] da Silva Rodrigues D.A., da Cunha C., do Espirito Santo D.R., de Barros A.L.C., Pereira A.R., de Queiroz Silva S., da Fonseca Santiago A., de Cassia Franco Afonso R.J. (2021). Removal of cephalexin and erythromycin antibiotics, and their resistance genes, by microalgae-bacteria consortium from wastewater treatment plant secondary effluents. Environ. Sci. Pollut. Res. Int..

[27] Gnida A., Felis E., Ziembinska-Buczynska A., Luczkiewicz A., Surmacz-Gorska J., Olanczuk-Neyman K. (2020). Evidence of mutations conferring resistance to clarithromycin in wastewater and activated sludge. 3 Biotech.

[28] Basturk I., Varank G., Murat-Hocaoglu S., Yazici-Guvenc S., Can-Güven E., Oktem-Olgun E.E., Canli O. (2021). Simultaneous degradation of cephalexin, ciprofloxacin, and clarithromycin from medical laboratory wastewater by electro-Fenton process. J. Environ. Chem. Eng..

[29] Kummerer K., al-Ahmad A., Mersch-Sundermann V. (2000). Biodegradability of some antibiotics, elimination of the genotoxicity and affection of wastewater bacteria in a simple test. Chemosphere.

[30] Brown K.D., Kulis J., Thomson B., Chapman T.H., Mawhinney D.B. (2006). Occurrence of antibiotics in hospital, residential, and dairy effluent, municipal wastewater, and the Rio Grande in New Mexico. Sci. Total Environ..

[31] Jones-Lepp T.L., Stevens R. (2007). Pharmaceuticals and personal care products in biosolids/sewage sludge: The interface between analytical chemistry and regulation. Anal. Bioanal. Chem..

[32] Jelić A., Gros M., Petrović M., Ginebreda A., Barceló D., Guasch H., Ginebreda A., Geiszinger A. (2012). Occurrence and Elimination of Pharmaceuticals During Conventional Wastewater Treatment. Emerging and Priority Pollutants in Rivers.

[33] Yu R., Wu Z. (2020). High adsorption for ofloxacin and reusability by the use of ZIF-8 for wastewater treatment. Microporous Mesoporous Mater..

[34] Rodrigues-Silva C., Porto R., dos Santos S., Schneider J., Rath S. (2019). Fluoroquinolones in Hospital Wastewater: Analytical Method, Occurrence, Treatment with Ozone and Residual Antimicrobial Activity Evaluation. J. Braz. Chem. Soc..

[35] Minato Y., Dawadi S., Kordus S.L., Sivanandam A., Aldrich C.C., Baughn A.D. (2018). Mutual potentiation drives synergy between trimethoprim and sulfamethoxazole. Nat. Commun..

[36] Eliopoulos G.M., Moellering R.C. (1982). Antibiotic synergism and antimicrobial combinations in clinical infections. Rev. Infect. Dis..

[37] Xu X., Xu L., Yuan G., Wang Y., Qu Y., Zhou M. (2018). Synergistic combination of two antimicrobial agents closing each other’s mutant selection windows to prevent antimicrobial resistance. Sci. Rep..

[38] Hegreness M., Shoresh N., Damian D., Hartl D., Kishony R. (2008). Accelerated evolution of resistance in multidrug environments. Proc. Natl. Acad. Sci. USA.

[39] Pena-Miller R., Lahnemann D., Schulenburg H., Ackermann M., Beardmore R. (2012). The optimal deployment of synergistic antibiotics: A control-theoretic approach. J. R. Soc. Interface.

[40] Chait R., Craney A., Kishony R. (2007). Antibiotic interactions that select against resistance. Nature.

[41] Uluseker C., Kaster K.M., Thorsen K., Basiry D., Shobana S., Jain M., Kumar G., Kommedal R., Pala-Ozkok I. (2021). A Review on Occurrence and Spread of Antibiotic Resistance in Wastewaters and in Wastewater Treatment Plants: Mechanisms and Perspectives. Front. Microbiol..

[42] Agerstrand M., Berg C., Bjorlenius B., Breitholtz M., Brunstrom B., Fick J., Gunnarsson L., Larsson D.G., Sumpter J.P., Tysklind M. (2015). Improving environmental risk assessment of human pharmaceuticals. Environ. Sci. Technol..

[43] Bengtsson-Palme J., Larsson D.G. (2016). Concentrations of antibiotics predicted to select for resistant bacteria: Proposed limits for environmental regulation. Environ. Int..

[44] Link M., von der Ohe P.C., Voss K., Schafer R.B. (2017). Comparison of dilution factors for German wastewater treatment plant effluents in receiving streams to the fixed dilution factor from chemical risk assessment. Sci. Total Environ..

[45] Freeling F., Alygizakis N.A., von der Ohe P.C., Slobodnik J., Oswald P., Aalizadeh R., Cirka L., Thomaidis N.S., Scheurer M. (2019). Occurrence and potential environmental risk of surfactants and their transformation products discharged by wastewater treatment plants. Sci. Total Environ..

[46] Thomaidis N.S., Gago-Ferrero P., Ort C., Maragou N.C., Alygizakis N.A., Borova V.L., Dasenaki M.E. (2016). Reflection of Socioeconomic Changes in Wastewater: Licit and Illicit Drug Use Patterns. Environ. Sci. Technol..

[47] Schymanski E.L., Jeon J., Gulde R., Fenner K., Ruff M., Singer H.P., Hollender J. (2014). Identifying small molecules via high resolution mass spectrometry: Communicating confidence. Environ. Sci. Technol..

[48] Mohammed Taha H., Aalizadeh R., Alygizakis N., Antignac J.P., Arp H.P.H., Bade R., Baker N., Belova L., Bijlsma L., Bolton E.E. (2022). The NORMAN Suspect List Exchange (NORMAN-SLE): Facilitating European and worldwide collaboration on suspect screening in high resolution mass spectrometry. Environ. Sci. Eur..

[49] Alygizakis N.A., Oswald P., Thomaidis N.S., Schymanski E.L., Aalizadeh R., Schulze T., Oswaldova M., Slobodnik J. (2019). NORMAN digital sample freezing platform: A European virtual platform to exchange liquid chromatography high resolution-mass spectrometry data and screen suspects in “digitally frozen” environmental samples. TrAC Trends Anal. Chem..

[50] Aalizadeh R., Alygizakis N.A., Schymanski E.L., Krauss M., Schulze T., Ibanez M., McEachran A.D., Chao A., Williams A.J., Gago-Ferrero P. (2021). Development and Application of Liquid Chromatographic Retention Time Indices in HRMS-Based Suspect and Nontarget Screening. Anal. Chem..

[51] Djoumbou-Feunang Y., Pon A., Karu N., Zheng J., Li C., Arndt D., Gautam M., Allen F., Wishart D.S. (2019). CFM-ID 3.0: Significantly Improved ESI-MS/MS Prediction and Compound Identification. Metabolites.

[52] Alygizakis N., Galani A., Rousis N.I., Aalizadeh R., Dimopoulos M.A., Thomaidis N.S. (2021). Change in the chemical content of untreated wastewater of Athens, Greece under COVID-19 pandemic. Sci. Total Environ..

[53] Chen X., Reynolds C.H. (2002). Performance of Similarity Measures in 2D Fragment-Based Similarity Searching:  Comparison of Structural Descriptors and Similarity Coefficients. J. Chem. Inf. Comput. Sci..

[54] von der Ohe P.C., Dulio V., Slobodnik J., De Deckere E., Kuhne R., Ebert R.U., Ginebreda A., De Cooman W., Schuurmann G., Brack W. (2011). A new risk assessment approach for the prioritization of 500 classical and emerging organic microcontaminants as potential river basin specific pollutants under the European Water Framework Directive. Sci. Total Environ..

[55] Dulio V., von der Ohe P.C. (2013). NORMAN Prioritisation Framework for Emerging Substances. http://www.norman-network.net/sites/default/files/norman_prioritisation_manual_15%20April2013_final_for_website.pdf.

[56] Aalizadeh R., von der Ohe P.C., Thomaidis N.S. (2017). Prediction of acute toxicity of emerging contaminants on the water flea Daphnia magna by Ant Colony Optimization-Support Vector Machine QSTR models. Environ. Sci. Process Impacts.

[57] Slobodnik J., Mrafkova L., Carere M., Ferrara F., Pennelli B., Schüürmann G., von der Ohe P.C. (2012). Identification of river basin specific pollutants and derivation of environmental quality standards: A case study in the Slovak Republic. TrAC Trends Anal. Chem..

